# Expression of concern: Novel magnetic iron oxide nanoparticles coated with sulfonated asphaltene as crude oil spill collectors

**DOI:** 10.1039/d5ra90044a

**Published:** 2025-04-14

**Authors:** Mahmood M. S. Abdullah, Hamad A. Al-Lohedan, Ayman M. Atta

**Affiliations:** a Surfactants Research Chair, Chemistry Department, College of Science, King Saud University Riyadh 11451 Saudi Arabia aatta@ksu.edu.sa hlohedan@ksu.edu.sa Mr.alhddad@gmail.com +966 114675998 +966 561557975; b Petroleum Application Department, Egyptian Petroleum Research Institute Nasr City 11727 Cairo Egypt +20 222747433 +20 222747917

## Abstract

Expression of concern for ‘Novel magnetic iron oxide nanoparticles coated with sulfonated asphaltene as crude oil spill collectors’ by Mahmood M. S. Abdullah *et al.*, *RSC Adv.*, 2016, **6**, 59242–59249, https://doi.org/10.1039/C6RA09651D.

*RSC Advances* is publishing this expression of concern in order to alert readers that concerns have been raised regarding the integrity of the FT-IR data in Fig. 1 and the TEM data in Fig. 4a.

An expression of concern will continue to be associated with the article until a conclusive outcome is reached.

Laura Fisher

4th April 2025

Executive Editor, *RSC Advances*

